# *catena*-Poly[[diaqua­cobalt(II)]-μ_2_-7-oxa­bicyclo­[2.2.1]heptane-2,3-dicarboxyl­ato-κ^4^ *O* ^2^,*O* ^3^,*O* ^7^:*O* ^2′^]

**DOI:** 10.1107/S1600536812000554

**Published:** 2012-01-14

**Authors:** Fan Zhang, Qiu-Yue Lin, Yong-Chang Wang, Ji-Du He

**Affiliations:** a Zhejiang Key Laboratory for Reactive Chemistry on Solid Surfaces, Institute of Physical Chemistry, Zhejiang Normal University, Jinhua, Zhejiang 321004, People’s Republic of China; b College of Chemistry and Life Science, Zhejiang Normal University, Jinhua 321004, Zhejiang, People’s Republic of China

## Abstract

The polymeric title complex, [Co(C_8_H_8_O_5_)(H_2_O)_2_]_*n*_ was synthesized by reaction of cobalt acetate with 7-oxabicyclo­[2,2,1]heptane-2,3-dicarb­oxy­lic anhydride. The Co^II^ ion is six-coordinated in a distorted octa­hedral environment, binding to two water O atoms, to the ether O atom of the bicyclo­heptane unit, to two carboxyl­ate O atoms from two different carboxyl­ate groups of the same anion and to one carboxyl­ate O atom from a symmetry-related anion. The bridging character of the dianion leads to the formation of ribbons along [001]. The ribbons are linked into a layered network parallel to (010) by several O—H⋯O hydrogen-bonding inter­actions involving the coordinating water mol­ecules as donors and the carboxyl­ate O atoms of neighbouring ribbons as acceptors. The crystal under investigation was an inversion twin.

## Related literature

For background to the applications of norcantharidin [systematic name: 7-oxabicyclo­(2.2.1)heptane-2,3-dicarb­oxy­lic anhydride], see: Yang *et al.* (2002 ▶). For the isotypic Cu analogue, see: Wang *et al.* (2009*a* ▶), and for a related Ni complex with monoclinic symmetry, see: Wang *et al.* (2009*b* ▶).
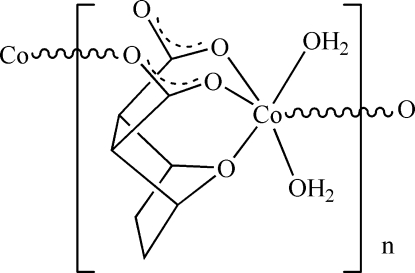


## Experimental

### 

#### Crystal data

[Co(C_8_H_8_O_5_)(H_2_O)_2_]
*M* *_r_* = 279.11
Orthorhombic, 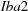

*a* = 10.3794 (10) Å
*b* = 18.983 (3) Å
*c* = 10.5021 (12) Å
*V* = 2069.3 (5) Å^3^
*Z* = 8
Mo *K*α radiation
μ = 1.68 mm^−1^
*T* = 296 K
0.22 × 0.15 × 0.10 mm

#### Data collection

Bruker APEXII area-detector diffractometer
Absorption correction: multi-scan (*SADABS*; Bruker, 2006 ▶) *T*_min_ = 0.742, *T*_max_ = 0.851
13174 measured reflections
1837 independent reflections
1821 reflections with *I* > 2σ(*I*)
*R*_int_ = 0.025

#### Refinement

*R*[*F* ^2^ > 2σ(*F* ^2^)] = 0.029
*wR*(*F* ^2^) = 0.088
*S* = 1.00
1837 reflections
146 parameters
7 restraints
H-atom parameters constrained
Δρ_max_ = 0.32 e Å^−3^
Δρ_min_ = −0.77 e Å^−3^
Absolute structure: Flack (1983 ▶), 860 Friedel pairs
Flack parameter: 0.12 (3)

### 

Data collection: *APEX2* (Bruker, 2006 ▶); cell refinement: *SAINT* (Bruker, 2006 ▶); data reduction: *SAINT*; program(s) used to solve structure: *SHELXS97* (Sheldrick, 2008 ▶); program(s) used to refine structure: *SHELXL97* (Sheldrick, 2008 ▶); molecular graphics: *DIAMOND* (Brandenburg, 2006 ▶); software used to prepare material for publication: *SHELXL97*.

## Supplementary Material

Crystal structure: contains datablock(s) I, global. DOI: 10.1107/S1600536812000554/wm2580sup1.cif

Structure factors: contains datablock(s) I. DOI: 10.1107/S1600536812000554/wm2580Isup2.hkl

Additional supplementary materials:  crystallographic information; 3D view; checkCIF report

## Figures and Tables

**Table 1:** Hydrogen-bond geometry (Å, °)

*D*—H⋯*A*	*D*—H	H⋯*A*	*D*⋯*A*	*D*—H⋯*A*
O1*W*—H1*WA*⋯O1^i^	0.85	1.98	2.832 (3)	180
O2*W*—H2*WB*⋯O4^i^	0.85	1.96	2.811 (3)	180
O1*W*—H1*WB*⋯O4^ii^	0.85	1.95	2.800 (3)	180
O2*W*—H2*WA*⋯O3^iii^	0.85	1.86	2.708 (3)	180

Symmetry codes: (i) 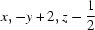; (ii) 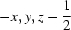; (iii) 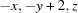.

**Fig. 1.**
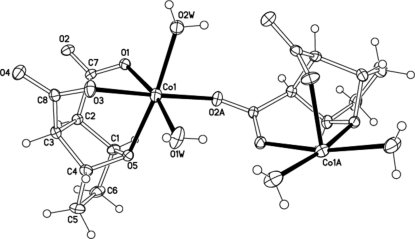
The coordination of the Co2+ ion with atom-labelling scheme and displacement ellipsoids drawn at the 30% probability level. [[Symmetry code (A) x, -y+2, z-1/2].

**Fig. 2.**
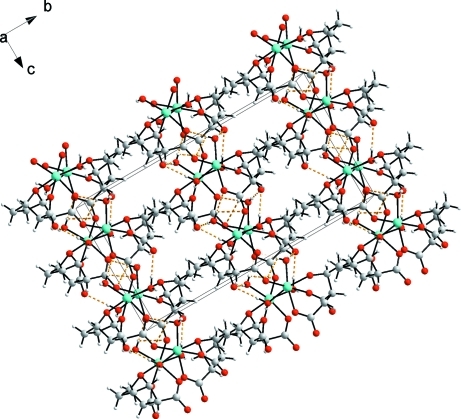
The one-dimensional polymeric structure of the title complex along [001] with hydrogen bonding interactions (dotted lines).

## supplementary crystallographic information

### Comment

7-oxabicyclo[2,2,1]heptane-2,3-dicarboxylic anhydride (norcantharidin), a traditional Chinese drug, has great anti-cancer activity. The coordination chemistry of cobalt has been important in biology mainly because of coenzyme B_12_ (Yang *et al.*, 2002). Therefore studying the combination of norcantharidin and cobalt seemed interesting. In this communication, the polymeric title complex, [Co(C_8_H_8_O_5_)(H_2_O)_2_]_n_ is reported.

The isostructural cooper complex (Wang *et al.*, 2009*a*) and a similar nickel complex with monoclinic symmetry (Wang *et al.*, 2009*b*) of demethylcantharate have been reported previously. The coordination of the Co^2+^ ion in the title complex is shown in Fig. 1. The Co^2+^ ion is six-coordinated in a distorted octahedral coordination mode, binding to two water O atoms, to the bridging O atom of the bicycloheptane unit, to two carboxylate O atoms from different carboxylate groups and to one carboxylate O atom from a symmetry-related bridging anion. This leads to the formation of ribbons extending along [001] (Fig. 2).

As also shown in Fig. 2, the ribbons are linked into a two-dimensional network parallel to (010) by several O—H···O hydrogen-bonding interactions involving the coordinating water molecules as donors and the carboxylate O atoms of neighbouring ribbons as acceptors.

### Experimental

A mixture of 0.5 mmol norcantharidin, 0.5 mmol cobalt acetate and 15 mL distilled water was sealed in a 25 mL Teflon-lined stainless vessel and heated at 443 K for 3 d, then cooled slowly to room temperature. The solution was filtered and block red crystals were obtained.

### Refinement

H atoms bonded to C atoms were positioned geometrically and refined using a riding model [aliphatic tertiary carbon C—H = 0.98 Å, aliphatic secondary carbon C—H = 0.97 Å, both with *U*_iso_(H) = 1.2*U*_eq_(C)]. The H atoms bonded to the O atoms were located in a difference Fourier map and refined with O—H distance restraints of 0.85 (1) Å and *U*_iso_(H) = 1.5*U*_eq_(O). The crystal under investigation was an inversion twin with a ratio of 0.88 (3):0.12 (3).

### Crystal data

**Table:**

[Co(C_8_H_8_O_5_)(H_2_O)_2_]	*F*(000) = 1144
*M**_r_* = 279.11	*D*_x_ = 1.792 Mg m^−^^3^
Orthorhombic, *I**b**a*2	Mo *K*α radiation, λ = 0.71073 Å
Hall symbol: I 2 -2c	Cell parameters from 9954 reflections
*a* = 10.3794 (10) Å	θ = 2.2–25.0°
*b* = 18.983 (3) Å	µ = 1.68 mm^−^^1^
*c* = 10.5021 (12) Å	*T* = 296 K
*V* = 2069.3 (5) Å^3^	Block, red
*Z* = 8	0.22 × 0.15 × 0.10 mm

### Data collection

**Table:**

Bruker APEXII area-detector diffractometer	1837 independent reflections
Radiation source: fine-focus sealed tube	1821 reflections with *I* > 2σ(*I*)
graphite	*R*_int_ = 0.025
ω scans	θ_max_ = 25.0°, θ_min_ = 2.2°
Absorption correction: multi-scan (*SADABS*; Bruker, 2006)	*h* = −12→9
*T*_min_ = 0.742, *T*_max_ = 0.851	*k* = −22→22
13174 measured reflections	*l* = −12→12

### Refinement

**Table:**

Refinement on *F*^2^	Secondary atom site location: difference Fourier map
Least-squares matrix: full	Hydrogen site location: inferred from neighbouring sites
*R*[*F*^2^ > 2σ(*F*^2^)] = 0.029	H-atom parameters constrained
*wR*(*F*^2^) = 0.088	*w* = 1/[σ^2^(*F*_o_^2^) + (0.0734*P*)^2^ + 1.6545*P*] where *P* = (*F*_o_^2^ + 2*F*_c_^2^)/3
*S* = 1.00	(Δ/σ)_max_ = 0.001
1837 reflections	Δρ_max_ = 0.32 e Å^−^^3^
146 parameters	Δρ_min_ = −0.77 e Å^−^^3^
7 restraints	Absolute structure: Flack (1983), 860 Friedel pairs
Primary atom site location: structure-invariant direct methods	Flack parameter: 0.12 (3)

### Special details

**Table:**

Geometry. All e.s.d.'s (except the e.s.d. in the dihedral angle between two l.s. planes) are estimated using the full covariance matrix. The cell e.s.d.'s are taken into account individually in the estimation of e.s.d.'s in distances, angles and torsion angles; correlations between e.s.d.'s in cell parameters are only used when they are defined by crystal symmetry. An approximate (isotropic) treatment of cell e.s.d.'s is used for estimating e.s.d.'s involving l.s. planes.
Refinement. Refinement of *F*^2^ against ALL reflections. The weighted *R*-factor *wR* and goodness of fit *S* are based on *F*^2^, conventional *R*-factors *R* are based on *F*, with *F* set to zero for negative *F*^2^. The threshold expression of *F*^2^ > σ(*F*^2^) is used only for calculating *R*-factors(gt) *etc*. and is not relevant to the choice of reflections for refinement. *R*-factors based on *F*^2^ are statistically about twice as large as those based on *F*, and *R*- factors based on ALL data will be even larger.

### Fractional atomic coordinates and isotropic or equivalent isotropic displacement parameters (Å^2^)

**Table:**

	*x*	*y*	*z*	*U*_iso_*/*U*_eq_	
Co1	0.23966 (4)	0.966647 (19)	0.74932 (8)	0.02813 (16)	
O1W	0.1588 (3)	0.91672 (15)	0.5806 (2)	0.0429 (6)	
H1WA	0.2080	0.9431	0.5369	0.064*	
H1WB	0.0843	0.8987	0.5727	0.064*	
O1	0.3221 (2)	0.99510 (11)	0.9348 (2)	0.0248 (4)	
O2W	0.1226 (2)	1.06142 (12)	0.7759 (2)	0.0374 (6)	
H2WA	0.0483	1.0731	0.8026	0.056*	
H2WB	0.1121	1.0858	0.7089	0.056*	
O2	0.3530 (2)	0.96635 (11)	1.1378 (2)	0.0257 (5)	
O3	0.1138 (2)	0.90091 (11)	0.8609 (2)	0.0303 (5)	
O4	0.0868 (2)	0.85747 (12)	1.0548 (2)	0.0309 (5)	
O5	0.37724 (17)	0.87420 (9)	0.76172 (19)	0.0215 (4)	
C1	0.4783 (3)	0.87921 (15)	0.8584 (3)	0.0242 (6)	
H1	0.5346	0.9202	0.8480	0.029*	
C2	0.4010 (3)	0.87971 (14)	0.9824 (3)	0.0201 (6)	
H2	0.4522	0.8587	1.0510	0.024*	
C3	0.2838 (3)	0.82969 (15)	0.9480 (3)	0.0217 (6)	
H3	0.2852	0.7878	1.0023	0.026*	
C4	0.3196 (3)	0.80965 (14)	0.8104 (3)	0.0258 (6)	
H4	0.2465	0.7927	0.7597	0.031*	
C5	0.4367 (4)	0.75899 (17)	0.8103 (4)	0.0389 (8)	
H5A	0.4272	0.7223	0.8738	0.047*	
H5B	0.4492	0.7376	0.7273	0.047*	
C6	0.5475 (3)	0.80918 (18)	0.8437 (3)	0.0351 (7)	
H6A	0.5895	0.7953	0.9224	0.042*	
H6B	0.6110	0.8110	0.7760	0.042*	
C7	0.3557 (2)	0.95301 (15)	1.0216 (3)	0.0179 (6)	
C8	0.1514 (3)	0.86588 (14)	0.9561 (3)	0.0211 (6)	

### Atomic displacement parameters (Å^2^)

**Table:**

	*U*^11^	*U*^22^	*U*^33^	*U*^12^	*U*^13^	*U*^23^
Co1	0.0293 (2)	0.0306 (3)	0.0245 (2)	−0.00025 (14)	0.0007 (2)	0.0048 (2)
O1W	0.0397 (14)	0.0645 (17)	0.0247 (11)	−0.0249 (12)	−0.0039 (11)	0.0043 (12)
O1	0.0329 (11)	0.0223 (10)	0.0191 (9)	0.0030 (8)	0.0000 (9)	0.0004 (8)
O2W	0.0309 (12)	0.0419 (12)	0.0394 (14)	0.0150 (10)	0.0098 (10)	0.0125 (10)
O2	0.0236 (12)	0.0331 (10)	0.0203 (10)	0.0057 (8)	−0.0002 (9)	−0.0052 (7)
O3	0.0186 (10)	0.0394 (12)	0.0329 (12)	0.0013 (9)	0.0023 (9)	0.0144 (10)
O4	0.0272 (11)	0.0399 (12)	0.0257 (11)	0.0020 (9)	0.0057 (9)	0.0035 (9)
O5	0.0231 (9)	0.0226 (9)	0.0187 (10)	0.0008 (7)	0.0006 (8)	−0.0003 (8)
C1	0.0195 (13)	0.0282 (14)	0.0249 (15)	0.0029 (11)	0.0012 (12)	−0.0046 (11)
C2	0.0196 (13)	0.0224 (14)	0.0183 (13)	0.0023 (11)	−0.0012 (11)	−0.0008 (11)
C3	0.0240 (13)	0.0184 (13)	0.0227 (14)	−0.0009 (12)	−0.0005 (12)	0.0036 (11)
C4	0.0310 (16)	0.0205 (13)	0.0259 (14)	−0.0007 (11)	−0.0011 (13)	−0.0034 (11)
C5	0.051 (2)	0.0250 (15)	0.0408 (18)	0.0140 (14)	0.0077 (17)	−0.0045 (13)
C6	0.0303 (16)	0.0438 (17)	0.0312 (17)	0.0170 (14)	0.0038 (15)	−0.0048 (14)
C7	0.0125 (12)	0.0237 (13)	0.0174 (14)	−0.0020 (10)	−0.0001 (11)	−0.0046 (11)
C8	0.0200 (14)	0.0196 (13)	0.0238 (14)	−0.0038 (10)	−0.0011 (12)	−0.0001 (11)

### Geometric parameters (Å, °)

**Table:**

Co1—O2^i^	2.091 (2)	C1—C6	1.519 (4)
Co1—O3	2.154 (2)	C1—C2	1.529 (4)
Co1—O1W	2.178 (3)	C1—H1	0.9800
Co1—O2W	2.188 (2)	C2—C7	1.525 (4)
Co1—O1	2.194 (2)	C2—C3	1.585 (4)
Co1—O5	2.2664 (18)	C2—H2	0.9800
O1W—H1WA	0.8500	C3—C8	1.539 (4)
O1W—H1WB	0.8500	C3—C4	1.540 (4)
O1—C7	1.262 (4)	C3—H3	0.9800
O2W—H2WA	0.8501	C4—C5	1.550 (4)
O2W—H2WB	0.8499	C4—H4	0.9800
O2—C7	1.246 (4)	C5—C6	1.534 (5)
O2—Co1^ii^	2.091 (2)	C5—H5A	0.9700
O3—C8	1.262 (4)	C5—H5B	0.9700
O4—C8	1.244 (4)	C6—H6A	0.9700
O5—C4	1.456 (3)	C6—H6B	0.9700
O5—C1	1.462 (4)		
			
O2^i^—Co1—O3	176.84 (9)	C1—C2—C3	101.8 (2)
O2^i^—Co1—O1W	91.46 (10)	C7—C2—H2	109.9
O3—Co1—O1W	87.54 (10)	C1—C2—H2	109.9
O2^i^—Co1—O2W	83.31 (9)	C3—C2—H2	109.9
O3—Co1—O2W	94.03 (8)	C8—C3—C4	112.2 (2)
O1W—Co1—O2W	104.34 (11)	C8—C3—C2	113.9 (2)
O2^i^—Co1—O1	97.37 (9)	C4—C3—C2	100.2 (2)
O3—Co1—O1	84.03 (9)	C8—C3—H3	110.0
O1W—Co1—O1	168.35 (9)	C4—C3—H3	110.0
O2W—Co1—O1	84.31 (9)	C2—C3—H3	110.0
O2^i^—Co1—O5	98.56 (8)	O5—C4—C3	102.7 (2)
O3—Co1—O5	84.39 (7)	O5—C4—C5	101.5 (2)
O1W—Co1—O5	87.28 (10)	C3—C4—C5	110.1 (2)
O2W—Co1—O5	168.22 (10)	O5—C4—H4	113.8
O1—Co1—O5	83.92 (8)	C3—C4—H4	113.8
Co1—O1W—H1WA	87.2	C5—C4—H4	113.8
Co1—O1W—H1WB	127.2	C6—C5—C4	101.7 (2)
H1WA—O1W—H1WB	137.0	C6—C5—H5A	111.4
C7—O1—Co1	126.40 (17)	C4—C5—H5A	111.4
Co1—O2W—H2WA	139.4	C6—C5—H5B	111.4
Co1—O2W—H2WB	114.5	C4—C5—H5B	111.4
H2WA—O2W—H2WB	90.8	H5A—C5—H5B	109.3
C7—O2—Co1^ii^	133.2 (2)	C1—C6—C5	102.2 (3)
C8—O3—Co1	123.30 (19)	C1—C6—H6A	111.3
C4—O5—C1	96.1 (2)	C5—C6—H6A	111.3
C4—O5—Co1	114.38 (16)	C1—C6—H6B	111.3
C1—O5—Co1	116.19 (14)	C5—C6—H6B	111.3
O5—C1—C6	102.2 (2)	H6A—C6—H6B	109.2
O5—C1—C2	102.4 (2)	O2—C7—O1	124.9 (3)
C6—C1—C2	109.8 (2)	O2—C7—C2	117.2 (2)
O5—C1—H1	113.7	O1—C7—C2	117.9 (2)
C6—C1—H1	113.7	O4—C8—O3	124.1 (3)
C2—C1—H1	113.7	O4—C8—C3	118.0 (3)
C7—C2—C1	113.4 (2)	O3—C8—C3	117.8 (3)
C7—C2—C3	111.8 (2)		
			
O2^i^—Co1—O1—C7	−134.4 (2)	C7—C2—C3—C4	122.2 (2)
O3—Co1—O1—C7	48.5 (2)	C1—C2—C3—C4	0.9 (3)
O1W—Co1—O1—C7	4.6 (6)	C1—O5—C4—C3	57.7 (2)
O2W—Co1—O1—C7	143.1 (2)	Co1—O5—C4—C3	−64.7 (2)
O5—Co1—O1—C7	−36.5 (2)	C1—O5—C4—C5	−56.1 (2)
O1W—Co1—O3—C8	131.9 (2)	Co1—O5—C4—C5	−178.54 (17)
O2W—Co1—O3—C8	−123.9 (2)	C8—C3—C4—O5	85.4 (3)
O1—Co1—O3—C8	−40.0 (2)	C2—C3—C4—O5	−35.8 (3)
O5—Co1—O3—C8	44.4 (2)	C8—C3—C4—C5	−167.1 (2)
O2^i^—Co1—O5—C4	−167.10 (18)	C2—C3—C4—C5	71.7 (3)
O3—Co1—O5—C4	11.75 (19)	O5—C4—C5—C6	34.5 (3)
O1W—Co1—O5—C4	−76.03 (19)	C3—C4—C5—C6	−73.8 (3)
O2W—Co1—O5—C4	94.6 (4)	O5—C1—C6—C5	−35.0 (3)
O1—Co1—O5—C4	96.32 (19)	C2—C1—C6—C5	73.2 (3)
O2^i^—Co1—O5—C1	82.24 (18)	C4—C5—C6—C1	0.3 (3)
O3—Co1—O5—C1	−98.90 (18)	Co1^ii^—O2—C7—O1	27.9 (4)
O1W—Co1—O5—C1	173.31 (19)	Co1^ii^—O2—C7—C2	−151.0 (2)
O2W—Co1—O5—C1	−16.1 (5)	Co1—O1—C7—O2	−149.7 (2)
O1—Co1—O5—C1	−14.33 (18)	Co1—O1—C7—C2	29.1 (3)
C4—O5—C1—C6	57.0 (3)	C1—C2—C7—O2	−144.6 (3)
Co1—O5—C1—C6	178.01 (18)	C3—C2—C7—O2	101.0 (3)
C4—O5—C1—C2	−56.8 (2)	C1—C2—C7—O1	36.5 (3)
Co1—O5—C1—C2	64.2 (2)	C3—C2—C7—O1	−77.9 (3)
O5—C1—C2—C7	−86.2 (3)	Co1—O3—C8—O4	142.1 (2)
C6—C1—C2—C7	165.7 (2)	Co1—O3—C8—C3	−39.6 (3)
O5—C1—C2—C3	34.1 (3)	C4—C3—C8—O4	149.2 (3)
C6—C1—C2—C3	−74.0 (3)	C2—C3—C8—O4	−97.8 (3)
C7—C2—C3—C8	2.2 (3)	C4—C3—C8—O3	−29.2 (3)
C1—C2—C3—C8	−119.1 (2)	C2—C3—C8—O3	83.8 (3)

Symmetry codes: (i) *x*, −*y*+2, *z*−1/2; (ii) *x*, −*y*+2, *z*+1/2.

### Hydrogen-bond geometry (Å, °)

**Table:**

*D*—H···*A*	*D*—H	H···*A*	*D*···*A*	*D*—H···*A*
O1W—H1WA···O1^i^	0.85	1.98	2.832 (3)	180
O2W—H2WB···O4^i^	0.85	1.96	2.811 (3)	180
O1W—H1WB···O4^iii^	0.85	1.95	2.800 (3)	180
O2W—H2WA···O3^iv^	0.85	1.86	2.708 (3)	180

Symmetry codes: (i) *x*, −*y*+2, *z*−1/2; (iii) −*x*, *y*, *z*−1/2; (iv) −*x*, −*y*+2, *z*.
